# Cytoprotective Metal–Phenolic Network Sporulation to Modulate Microalgal Mobility and Division

**DOI:** 10.1002/advs.202308026

**Published:** 2023-11-28

**Authors:** Xiaojie Li, Hai Liu, Zhixing Lin, Joseph J. Richardson, Weiying Xie, Feng Chen, Wei Lin, Frank Caruso, Jiajing Zhou, Bin Liu

**Affiliations:** ^1^ Shenzhen Key Laboratory of Marine Microbiome Engineering Shenzhen Key Laboratory of Food Nutrition and Health Institute for Advanced Study College of Chemistry and Environmental Engineering Shenzhen University Shenzhen 518060 China; ^2^ College of Biomass Science and Engineering Key Laboratory of Leather Chemistry and Engineering of Ministry of Education National Engineering Laboratory for Clean Technology of Leather Manufacture Sichuan University Chengdu 610065 China; ^3^ Department of Chemical Engineering The University of Melbourne Parkville Victoria 3010 Australia; ^4^ Department of Chemical and Environmental Engineering RMIT University Melbourne Victoria 3000 Australia

**Keywords:** biohybrids, cell encapsulation, interfaces, metal‐organic coating, surface chemistry

## Abstract

Synthetic cell exoskeletons created from abiotic materials have attracted interest in materials science and biotechnology, as they can regulate cell behavior and create new functionalities. Here, a facile strategy is reported to mimic microalgal sporulation with on‐demand germination and locomotion via responsive metal–phenolic networks (MPNs). Specifically, MPNs with tunable thickness and composition are deposited on the surface of microalgae cells via one‐step coordination, without any loss of cell viability or intrinsic cell photosynthetic properties. The MPN coating keeps the cells in a dormant state, but can be disassembled on‐demand in response to environmental pH or chemical stimulus, thereby reviving the microalgae within 1 min. Moreover, the artificial sporulation of microalgae resulted in resistance to environmental stresses (e.g., metal ions and antibiotics) akin to the function of natural sporulation. This strategy can regulate the life cycle of complex cells, providing a synthetic strategy for designing hybrid microorganisms.

## Graphical abstract

Controlling microbial behavior is important for optimizing metabolite production and stress resistance for advanced biotechnologies. Microalgae armored with artificial metal–phenolic networks retain their intrinsic photosynthetic properties, and this coating modulates the cell life cycle and microbial motility and provides resilience to environmental stresses.

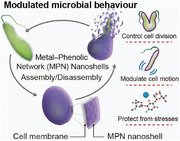

## 1 Introduction

Utilizing microorganisms as cellular factories has attracted significant scientific and industrial interest for the production of proteins,^[^ ^1^ ^]^ biofuels,^[^ ^2^, ^3^, ^4^ ^]^ and bioplastics^[^ ^5^ ^]^ in various fields including pharmaceuticals,^[^ ^6^, ^7^, ^8^, ^9^ ^]^ environmental remediation,^[^ ^10^, ^11^, ^12^ ^]^ and bioenergy.^[^ ^13^, ^14^, ^15^, ^16^ ^]^ Photosynthetic microalgae have emerged as compelling systems for converting carbon dioxide (CO_2_) into a vast array of biochemicals, especially in the context of carbon neutrality.^[^ ^17^, ^18^ ^]^ Regulating their behavior, e.g., sporulation and germination, is essential to facilitate the production of high‐value microalgal metabolites (e.g., lipids, proteins, and pigments) and to protect against biotic and abiotic stresses.^[^ ^19^ ^]^ To this end, genetic engineering and environmental manipulation with bioreactors have been employed to either develop microalgal strains with desired traits or regulate their life cycle.^[^ ^20^, ^21^, ^22^, ^23^, ^24^ ^]^ However, genetic modification relies on the engineering of cellular pathways, which is labor intensive and can be complex, while batch fermentation with bioreactors is energy and resource intensive. Therefore, a simple and effective method is needed for modulating microalgal growth and behavior to advance the utilization of controllable cellular factories.

Engineering the cell–environment interface offers promise for modulating cellular behavior in a dynamic way.^[^ ^25^, ^26^ ^]^ The spatially complex and customized environments enabled by materials support the high diversity of microbiota and mediate the essential biogeochemical cycling. Several strategies have been developed to engineer the surface of mammalian cells, bacteria, and fungi for cytoprotection and controlling cell division. For example, bioinspired silica,^[^ ^27^, ^28^, ^29^, ^30^ ^]^ metal–organic frameworks,^[^ ^31^, ^32^, ^33^, ^34^ ^]^ and polymer materials^[^ ^35^, ^36^, ^37^, ^38^, ^39^ ^]^ have been used to improve the stability and mechanical strength of microorganisms. Amorphous metal–phenolic networks (MPNs) have recently been introduced as a platform for engineering the surfaces of microorganisms^[^ ^40^, ^41^, ^42^, ^43^, ^44^, ^45^ ^]^ due to their advantages such as high biocompatibility, tunable mechanical strength, versatile functionalization, and rapid synthesis routes.^[^ ^46^, ^47^, ^48^ ^]^ Our previous work reported the use of MPNs to endow yeast with improved stress tolerance;^[^ ^49^ ^]^ however, controlling microbial motility along with germination and protection have not been demonstrated with MPNs, and are properties that are relatively unexplored in cell cytoprotection research.

**Figure 1:**
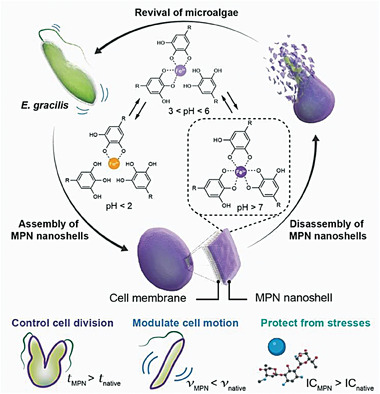
Schematic illustration showing the engineering of artificial microalgal sporulation and controlled cell motion via MPN coatings. *t*, generation time of microalgae; *v*, motion speed of microalgae; IC, inhibitory concentration of stimulus.

Here, we present an effective and straightforward approach for engineering the artificial microbial sporulation of microalgae, achieving controlled germination and locomotion with MPN nanoshells. Specifically, the surface of the microalgae was armored with amorphous MPN coatings through the one‐step coordination of polyphenols (e.g., tannic acid, TA) and metal ions (e.g., Fe^3+^) in a biological buffer (**Figure** **1**). The thickness and composition of the resulting MPN nanoshells could be adjusted by repeating the deposition process or changing the precursors. The MPN coating imparted no adverse effects on the photosynthetic behavior of the coated microalgae and had minimal toxicity, while still significantly modulating the cell division rate. Importantly, the MPN nanoshells could disassemble in acidic environments or under chemical stimuli (e.g., in the presence of a chelator), enabling the rapid breakdown (< 1 min) of the artificial spore (MPN) coating and subsequent revival of the dormant microalgae. Notably, the on–off sporulation governed the locomotion of the microalgae, a feat relatively unexplored in this field. Furthermore, the coated microalgae exhibited resilience to environmental stresses, such as toxic metal ions and antibiotics. This MPN‐mediated strategy not only regulates the cell behavior of microalgae in a controlled manner but also offers a synthetic approach for designing hybrid living systems for emerging biotechnologies.

## 2 Results and Discussion

**Figure 2:**
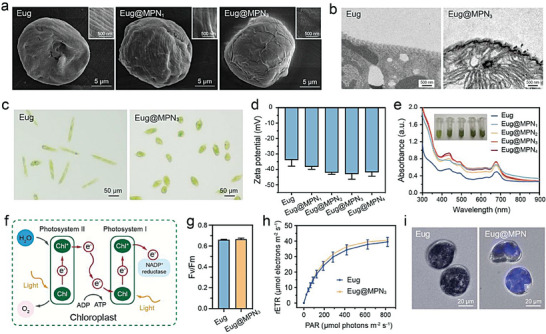
MPN‐encapsulated microalgae. a) Scanning electron microscopy (SEM) images of native *E. gracilis*, Eug@MPN_1_, and Eug@MPN_3_. b) TEM images of the membrane morphology of native *E. gracilis* and Eug@MPN_3_. c) Optical microscopy images of native *E. gracilis* and Eug@MPN_3_. d) Zeta potential values and e) UV–vis absorption spectra of *E. gracilis* before and after different MPN coating cycles. Inset in e) shows the corresponding suspensions of *E. gracilis* before and after MPN coating: native *E. gracilis*, Eug@MPN_1_, Eug@MPN_2_, Eug@MPN_3_, and Eug@MPN_4_ (from left to right). f) Schematic of the light‐dependent reactions of photosynthesis. Chl, chlorophyll molecule; NADP^+^, nicotinamide adenine dinucleotide phosphate; ADP, adenosine diphosphate; ATP, adenosine triphosphate. g) Photosynthetic efficiency (Fv/Fm) and h) relative electron transport rate (rETR) of *E. gracilis* before and after MPN nanoshell formation. PAR represents photosynthetically active radiation. i) Fluorescence microscopy images of *E. gracilis* and Eug@MPN with fluorescently labeled MPN nanoshells.

We first investigated the formation of MPN nanoshells on a single microbe, *Euglena gracilis* (*E. gracilis* or Eug). *E. gracilis* is a spindle‐shaped unicellular microalga that lacks a cell wall,^[^ ^50^ ^]^ but has a thin and flexible pellicle membrane made of proteinaceous strips that are arranged in a helical pattern (**Figure** **2**a). By simply mixing TA and FeCl_3_ in the presence of *E. gracilis*, a smooth coordination‐based MPN film was uniformly deposited on the cell surface, resulting in *E. gracilis*@MPN (Eug@MPN). The striations on the cell surface, resulting from the proteinaceous strips, disappeared after three MPN coating cycles, suggesting that a sufficiently thick MPN coating was applied (Figure 2a; Figure S1, Supporting Information). Transmission electron microscopy (TEM) images confirmed that the MPN nanoshells on Eug@MPN_3_ had a thickness of 34.7 ± 12.4 nm, or ≈11–12 nm per layer (Figure 2b).^[^ ^51^ ^]^ Interestingly, the formation of MPN nanoshells transformed the *E. gracilis* in solution from spindle‐shaped to ellipsoidal due to the structural confinement effect of the MPN nanoshells (Figure 2c). The surface zeta potential of *E. gracilis* gradually shifted from −33.8 ± 4.1 mV to −41.8 ± 2.7 mV by repeating the MPN coating cycles, indicating the binding of the anionic TA to the cell surface (Figure 2d). The formation of MPN nanoshells was further confirmed by the darker cell suspension color and appearance of a ligand‐to‐metal charge transfer band between 500 and 600 nm in the UV–vis absorption spectra of the Eug@MPN suspension (Figure 2e). In addition, three different metal ions including Zr^4+^, Cu^2+^, and Zn^2+^ could successfully generate MPN nanoshells on *E. gracilis*, confirming the versatility of this microalgae coating approach (Figure S2, Supporting Information).

Photosynthesis is an essential process that generates energy to support microalgae growth. Specifically, photosystems capture light energy with chlorophyll molecules, which promotes electrons to a higher energy level and enables their transfer through an electron transport chain to drive photosynthesis (Figure 2f).^[^ ^52^ ^]^ Despite the color of the MPN nanoshells, chlorophyll fluorescence analysis revealed that MPN nanoshells assembled from Fe^3+^ or Zr^4+^ had negligible influence on the photosynthetic efficiency (the ratio of variable fluorescence to maximum chlorophyll fluorescence, Fv/Fm) or the relative electron transport rate (rETR) of *E. gracilis* (Figures 2g,h and S3). This confirmed that the MPN nanoshells did not affect the photosynthetic performance of *E. gracilis*. To further visualize the MPN nanoshells formed on the microalgae, a blue fluorescent dye *N*‐phenyl‐1‐naphthylamine was used to label TA through π interactions, which was then subsequently mixed with metal ions (e.g., Zr^4+^) to assemble fluorescent MPN nanoshells on *E. gracilis*.^[^ ^53^ ^]^ The fluorescently labeled MPN nanoshells showed uniform fluorescence on the surface of the *E. gracilis*, confirming the core–shell structure of Eug@MPN_3_ (Figure 2i).

**Figure 3:**
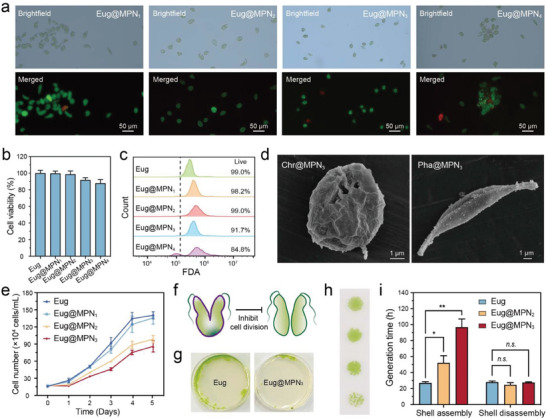
Biocompatibility and proliferation of MPN‐coated microalgae. a) Fluorescence microscopy images of Eug@MPN_1_, Eug@MPN_2_, Eug@MPN_3_, and Eug@MPN_4_. Cells were stained with FDA (live: green) and PI (dead: red). b) Cell viability of *E. gracilis* before and after MPN coating. c) Flow cytometry analysis of the viability of *E. gracilis* before and after MPN coating. Cells were stained with FDA. d) SEM images of Chr@MPN_3_ and Pha@MPN_3_. e) Cell growth profiles of *E. gracilis* before and after MPN formation. f) Schematic of the cell division suppression effect by MPN nanoshells. g) Effects of MPN nanoshells on the growth of *E. gracilis*. Cells were spread on agar plates and incubated for 10 days. h) Representative colonies obtained from native *E. gracilis* and *E. gracilis* coated with MPN nanoshells. From top to bottom: native *E. gracilis*, Eug@MPN_1_, Eug@MPN_2_, and Eug@MPN_3_. i) Control of *E. gracilis* cell division by MPN nanoshell formation and disassembly. Asterisk indicates a significant difference between treatments (* *p* < 0.001, ** *p* < 0.0001); *n.s*., no significant difference.

We examined the protective potential of the MPN nanoshells on the survival of the microalgae. To visualize the MPN‐coated cells and determine their viability, we employed fluorescein diacetate (FDA) and propidium iodide (PI) staining techniques, whereby live cells were identified with green fluorescence (FDA positive) and dead cells were identified with red fluorescence (PI positive). Predominately, green fluorescence was observed from MPN‐coated microalgal cells, indicating that most *E. gracilis* were viable and metabolically active after MPN coating (Figure 3a; Figure S4, Supporting Information). The cellular metabolic activity was also measured by an AlamarBlue assay, and the cell viability was 99.7 ± 2.9%, 98.7 ± 4.0%, 91.8 ± 2.6% and 87.9 ± 4.5% for Eug@MPN_1_, Eug@MPN_2_, Eug@MPN_3_, and Eug@MPN_4_, respectively (Figure 3b). The high biocompatibility of the coating processes was also confirmed by flow cytometry analysis (Figure 3c).

To show the versatility of MPN formation on different species of microalgae, we further assembled MPN nanoshells on spherical‐shaped *Chromochloris zofingiensis* (C. *zofingiensis* or Chr) that possess a rigid cell wall composed of polysaccharides and fusiform‐shaped *Phaeodactylum tricornutum* (*P. tricornutum* or Pha) that have a silica frustule. MPN nanoshells successfully formed on the surface of individual *C. zofingiensis* (Chr@MPN) and *P. tricornutum* (Pha@MPN) (Figure 3d), suggesting that MPN nanoshell formation is independent of the surface properties of the microalgae. Unlike *E. gracilis*, *C. zofingiensis* and *P. tricornutum* maintained their native cell morphology even after four MPN coating cycles due to their rigid nature (Figures S5 and S6, Supporting Information), and all of them showed high cell viability (> 95%) (Figures S7 and S8, Supporting Information). These above results demonstrate that MPN formation is a mild and highly biocompatible coating process for microalgae.

The high biocompatibility of the MPN nanoshells motivated us to further explore the impact of MPN nanoshells on the proliferative capacity of *E. gracilis*. We therefore evaluated the cell division by monitoring the cell growth of *E. gracilis* after MPN coating. The MPN‐coated *E. gracilis*, especially Eug@MPN_2_ and Eug@MPN_3_ exhibited a significantly delayed growth curve compared to native *E. gracilis* in the first three days after coating (Figure 3e). Similarly, MPN sporulation suppressed the colony formation of *E. gracilis* on agar plates with fewer and irregularly shaped colonies (Figures 3f–h; Figure S9, Supporting Information) that can be attributed to the reduced rate of cell division in individual cells. These findings are consistent with prior studies involving bacteria and yeast, as MPNs have been demonstrated to hinder the cell growth^[^ ^40^, ^49^ ^]^. Notably, native *E. gracilis* showed high‐frequency “swinging” motion due to phototaxis. This motion ceased upon the assembly of MPN nanoshells (Movie S1, Supporting Information). We further investigated the on‐demand disassembly of MPN nanoshells to recover cell division. After MPN coating, the generation time increased significantly from 26.9 ± 1.7 h for native *E. gracilis* to 52 ± 9.1 h for Eug@MPN_2_ or 96.8 ± 10.4 h for Eug@MPN_3_ (Figure 3i), indicating a notable retardation effect of MPN nanoshells on cell division. Upon 10 mm HCl treatment and removal of the MPN nanoshell, the generation time of Eug@MPN_2_ (24.6 ± 2.8 h) and Eug@MPN_3_ (27.5 ± 0.8 h) returned to a level comparable to that of the native *E. gracilis* (27.9 ± 1.6 h), effectively restoring the ability of the *E. gracilis* to divide naturally (Figure 3i).

**Figure 4:**
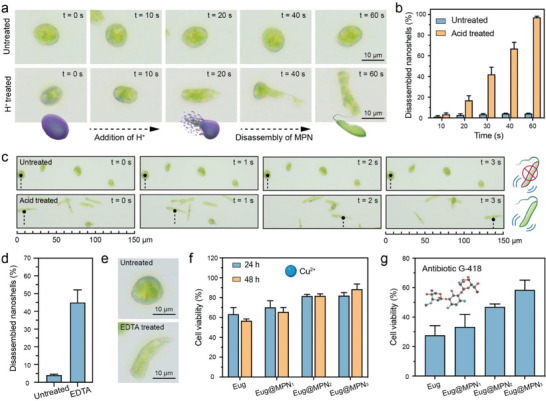
Mobility modulation and cytoprotection with MPN nanoshells. a) Optical microscopy images of the acid‐triggered disassembly process of MPN nanoshells from Eug@MPN_3_ within 60 s. b) Percentage of disassembled nanoshells from Eug@MPN_3_ during 60 s of acid treatment. c) Microscopy images of the motility of Eug@MPN_3_ before and after acid treatment. d) Percentage and e) microscopy images of Eug@MPN_3_ with disassembled nanoshells after incubation with 20 mm EDTA for 5 min. Cytoprotective ability of the MPN nanoshell against exposure to f) 0.2 mm CuCl_2_ for 24 and 48 h and g) 50 µm antibiotic G‐418 for 24 h.

We then investigated the manipulation of microalgal behavior before, during, and after MPN coating. The disassembly of MPN nanoshells from Eug@MPN_3_ commenced within 10 s of acid treatment, as evidenced by the morphological changes from round to elongated shape (**Figure** **4**a). This acid‐responsive disassembly process of the MPN nanoshells from Eug@MPN_3_ was observed in real time (Movie S2, Supporting Information). Specifically, most MPN nanoshells (97.1 ± 1.2%) underwent complete disassembly after 60 s of acid treatment due to the protonation of hydroxyl groups in TA molecules, which disrupts TA–Fe coordination (Figure 4b; Figure S10, Supporting Information). Notably, Eug@MPN_3_ could recover motion after 1 h of acid treatment (Movie S3, Supporting Information). After MPN nanoshell disassembly, the microalgae showed similar growth trends and pigment yield (e.g., chlorophyll a, chlorophyll b, and carotenoids) to control cells (Figure S11, Supporting Information), confirming the high biocompatibility of this on‐demand MPN assembly and disassembly process in maintaining cellular functions and pigment production. The locomotion of the microalgae was also monitored. While the MPN‐coated microalgae showed minimal mobility, those subjected to H^+^‐triggered germination exhibited the ability to swim at a velocity of 43 µm s^–1^ (Figure 4c). A similar responsive germination was observed with the addition of 20 mM ethylenediaminetetraacetic acid (EDTA), whereby 45.1 ± 7.1% Eug@MPN_3_ revived in 5 min, due to the strong affinity between EDTA and Fe^3+^ that triggered disassembly of the MPN nanoshells (Figure 4d,e).

The cytoprotective properties of the MPN nanoshells were examined against environmental stresses, such as exposure to heavy metal ions and antibiotics. Specifically, native *E. gracilis* was sensitive to Cu^2+^, as it leads to a significant inhibition on cell growth (Figure S12, Supporting Information). The 24 h and 48 h half‐maximal inhibitory concentration (IC_50_) values of Cu^2+^ were 0.34 ± 0.05 and 0.25 ± 0.02 mm, respectively (Figure S13, Supporting Information). In contrast, the cell viability increased with increasing thickness of the MPN nanoshells. The cell viability of *E. gracilis* increased from 56.6 ± 1.9% to 88.4 ± 5.2% with three MPN coatings after exposure to Cu^2+^ for 48 h (Figure 4f; Figure S14 and S15, Supporting Information), confirming the protective effects of MPN nanoshells against metal ions. Antibiotics were also investigated for their potential to inhibit the proliferation of microalgae, providing a means of controlling their population in various environments. After incubation with 50 µM antibiotic G‐418 for 24 h, Eug@MPN_1_ (33.3 ± 8.5%), Eug@MPN_2_ (46.9 ± 1.9%), and Eug@MPN_3_ (58.4 ± 6.7%) showed higher viabilities than native *E. gracilis* (27.6 ± 6.6%), indicating the protective nature of the MPN nanoshells (Figures 4g; Figures S16 and S17, Supporting Information).

## 3 Conclusion

In summary, this study presents a strategy for manipulating microalgal sporulation and motility using MPNs that are cryoprotective and stimuli‐responsive. The MPNs with tunable composition and thickness showed negligible impact on the intrinsic photosynthetic pathway and viability of the microalgae. The MPN coating disassembled in the presence of acidic environments or chemical stimuli, enabling the rapid breakdown of the nanoshell and rapid revival of the microalgae from a dormant state. Additionally, the artificial sporulation of microalgae exhibited increased tolerance to environmental stresses. This approach not only provides a means to regulate the life cycle of microalgae in a controlled manner but also offers a synthetic approach for designing living hybrid microalgae.

## Conflict of Interest

The authors declare no conflict of interest.

## Supporting information

Supporting Information

Supplemental Movie 1

Supplemental Movie 2

Supplemental Movie 3

## Data Availability

The data that support the findings of this study are available in the supplementary material of this article.
