# Supplementary material for: Cytoprotective Metal–Phenolic Network Sporulation to Modulate Microalgal Mobility and Division
Source: Adv Sci (Weinh). 2023 Nov 28;11(3):2308026. doi: 10.1002/advs.202308026 (PMC10797472; doi:10.1002/advs.202308026)
Supplement: Supplementary file 1 — Supporting Information [file ADVS-11-2308026-s004.pdf]

ADVANCED SCIENCE Open Access

Supporting Information

for *Adv. Sci.*, DOI 10.1002/advs.202308026

Cytoprotective Metal–Phenolic Network Sporulation to Modulate Microalgal Mobility and Division

Xiaojie Li, Hai Liu, Zhixing Lin, Joseph J. Richardson, Weiying Xie, Feng Chen, Wei Lin, Frank Caruso, Jiajing Zhou* and Bin Liu**

Supporting Information

Cytoprotective Metal–Phenolic Network Sporulation to Modulate Microalgal Mobility and Division

Xiaojie Li,[1,+] Hai Liu,[2,+] Zhixing Lin,[3] Joseph J. Richardson,[4] Weiying Xie,[1] Feng Chen,[1] Wei Lin,[2] Frank Caruso,*[3] Jiajing Zhou,*[2] and Bin Liu*[1]

1. Shenzhen Key Laboratory of Marine Microbiome Engineering, Institute for Advanced Study, Institute for Innovative Development of Food Industry, Shenzhen University, Shenzhen 518060, China

2. College of Biomass Science and Engineering, Key Laboratory of Leather Chemistry and Engineering of Ministry of Education, National Engineering Laboratory for Clean Technology of Leather Manufacture, Sichuan University, Chengdu 610065, China

3. Department of Chemical Engineering, The University of Melbourne, Parkville, Victoria, 3010 Australia

4. Department of Chemical and Environmental Engineering, RMIT University, Melbourne, Victoria, 3000 Australia

+ These authors contributed equally to this work.

Corresponding authors:

*(F. Caruso) Email: fcaruso@unimelb.edu.au

*(J. Zhou) Email: jjzhou@scu.edu.cn

*(B. Liu) Email: liubincoe@pku.edu.cn

Table of Contents

Experimental Procedures S3
Materials S3
Characterization S3
Algal Strains and Cultivation Methods S4
Assembly of Metal–Phenolic Network (MPN) Nanoshells on Microalgae S4
Assembly of MPN Nanoshells with Different Metal Ions S5
Fluorescence Labeling of MPN Nanoshells S5
Photosynthetic Performance Measurements S6
Biocompatibility Measurements of MPN Nanoshells S6
Disassembly of MPN Nanoshells on Microalgae S7
Control of Cell Division S7
Measurement of Pigment Yields S7
Modulation of Cell Motility S8
Protection from Copper-Induced Cytotoxicity Analysis S8
Protection from Antibiotic G-418 Analysis S8
Results and Discussion S9
References S18

Experimental Procedures

Materials

Tannic acid (TA) was obtained from Sigma-Aldrich (St. Louis, MO, USA). Iron(III) chloride hexahydrate ($FeCl_3 \cdot 6H_2O$), zirconium(IV) oxide chloride octahydrate ($ZrOCl_2 \cdot 8H_2O$), copper(II) chloride ($CuCl_2$), zinc chloride ($ZnCl_2$), 3-(*N*-morpholino)propanesulfonic acid (MOPS), dimethyl sulfoxide (DMSO), and ethylenediaminetetraacetic acid (EDTA) were purchased from Shanghai Macklin Biochemical Co., Ltd. (Shanghai, China). *N*-Phenyl-1-naphthylamine (NPN), propidium iodide (PI), and fluorescein diacetate (FDA) were obtained from Shanghai Aladdin Biochemical Technology Co., Ltd. (Shanghai, China). AlamarBlue cell viability reagent and glutaraldehyde (2.5%, EM grade) were purchased from Solarbio Life Science (Beijing, China). Agar and antibiotic G-418 were bought from Coolaber (Beijing, China). SYTOX Green was purchased from Thermo Fisher Scientific (Waltham, MA, USA). High-purity Milli-Q water with a resistivity of 18.2 MΩ cm was obtained from a Millipore Milli-Q purification system.

Characterization

Scanning electron microscopy (SEM) images were captured on a Thermo Apreo scanning electron microscope after sputter-coating with platinum. SEM samples were prepared by fixing with 2.5% glutaraldehyde at 4 °C overnight and dehydrating using a graded series of ethanol solutions. Then, a drop of sample dispersion was placed onto conductive cooper tapes, followed by drying overnight. UV–vis spectra were recorded on an Implen NanoPhotometer NP80. Zeta potential values were determined using a Brookhaven NanoBrook 90Plus particle size analyzer. Fluorescence microscopy images were obtained from a confocal laser scanning microscope (Leica STELLARIS 5). Cross-sectional transmission electron microscopy (TEM) images were obtained using a JEOL JEM F200 transmission electron microscope. Specimens were fixed

with 2.5% glutaraldehyde and dehydrated using a graded series of ethanol solution. The dehydrated microalgal cells were embedded in resin and ultrathin sectioned. Thin sections were further stained with uranyl acetate. Fluorescence spectroscopy measurements were performed on a BioTek Synergy H1 microplate reader.

Algal Strains and Cultivation Methods

Euglena gracilis (*E. gracilis* or Eug, CCAP 1224/5Z) were bought from the Culture Collection of Algae and Protozoa (CCAP; UK) and maintained in recommended liquid EG:JM medium (*Euglena gracilis* medium and Jaworski's medium were mixed at 1:1 ratio) at 23 °C and shaken at 150 rpm under continuous illumination of 50 μmol photons m^{-2} s^{-1}. For EG:JM agar plates cultivation, *E. gracilis* suspension was spread onto plates of EG:JM medium containing 15 g L^{-1} agar, which was incubated at 23 °C under continuous illumination of 50 μmol photons m^{-2} s^{-1}.

Chromochloris zofingiensis (*C. zofingiensis* or Chr, ATCC 30412) were obtained from the American Type Culture Collection (ATCC; USA) and grown in BG-11 liquid medium at 23 °C and shaken at 150 rpm under continuous illumination of 50 μmol photons m^{-2} s^{-1}.

Phaeodactylum tricornutum (*P. tricornutum* or Pha, UTEX 646) were purchased from UTEX Culture Collection of Algae (USA) and cultured in modification of Lewin's marine diatom (LDM) medium at 23 °C and shaken at 150 rpm under continuous illumination of 50 μmol photons m^{-2} s^{-1}.

Assembly of Metal–Phenolic Network (MPN) Nanoshells on Microalgae

E. gracilis in the exponential growth were collected by centrifuge (2000 *g*, 2 min) and resuspended in H_2O to reach optical density at 680 nm (OD_{680}) of 1.5. Then, 10 μL of TA (12 mM) and 10 μL of $FeCl_3 \cdot 6H_2O$ (12 mM) were successively and rapidly added to 480 μL of *E. gracilis* suspension with 10 s vortex mixing between the additions. Then, 500 μL of MOPS

buffer (20 mM, pH 7–8) was added to mixture to raise the pH, resulting in the formation of MPN nanoshells. Residual TA and $FeCl_3$ were removed by centrifugation (2000 *g*, 2 min) and washed with H_2O three times. The MPN nanoshell formation process was repeated 1–4 cycles to produce Eug@MPN$_1$, Eug@MPN$_2$, Eug@MPN$_3$, and Eug@MPN$_4$, respectively.

Assembly of MPN Nanoshells with Different Metal Ions

Eug@MPN$_3$-Zr was prepared using the same method as that described in *Assembly of Metal–Phenolic Network (MPN) Nanoshells on Microalgae* except that $FeCl_3 \cdot 6H_2O$ (12 mM) was replaced with $ZrOCl_2 \cdot 8H_2O$ (12 mM).

Eug@MPN$_3$-Cu was prepared using the same method as that described in *Assembly of Metal–Phenolic Network (MPN) Nanoshells on Microalgae* except that $FeCl_3 \cdot 6H_2O$ (12 mM) was replaced with $CuCl_2$ (12 mM).

Eug@MPN$_3$-Zn was prepared using the same method as that described in *Assembly of Metal–Phenolic Network (MPN) Nanoshells on Microalgae* except that $FeCl_3 \cdot 6H_2O$ (12 mM) was replaced with $ZnCl_2$ (12 mM).

Fluorescence Labeling of MPN Nanoshells

The fluorescence labeling of MPN nanoshells was conducted according to the procedures reported previously.[1] In brief, 10 μL of TA (12 mM), 2 μL of NPN (2 mg mL^{-1}; dissolved in DMSO), and 10 μL of $ZrOCl_2 \cdot 8H_2O$ (12 mM) were sequentially added to 480 μL of microalgal cell suspension ($OD_{680} = 1.5$) with vigorous vortex for 10 s. The pH of the suspension was raised and stabilized by adding 0.5 mL of MOPS buffer (20 mM, pH 7.5). Unreacted TA, NPN, and $ZrOCl_2 \cdot 8H_2O$ were removed by washing with H_2O three times. The fluorescence-labeled MPN was observed with an Olympus IX73 inverted fluorescence microscope and confocal microscopy.

Photosynthetic Performance Measurements

The photosynthetic performance was measured using a photosynethesis yield analyzer (WATER-PAM-II chlorophyll fluorometer, Walz, Germany). Briefly, cells were dark-adapted (i.e., kept in complete darkness) for 20 min and then the steady-state chlorophyll fluorescence (F_0) and maximum chlorophyll fluorescence (F_m) were obatiend by using a modulated light and a brief pulse of saturating light, respectively. Thereafter, the photosynthetic efficiency (the ratio of variable fluorescence to maximum fluorescence, F_v/F_m) was calculated as:[2]

$$F_v/F_m = (F_m - F_0)/F_m \quad (1)$$

In addition, the relative photosynthetic electron transport rate (rETR) of light-dependent reactions was measured by photosynthetically active radiation (PAR) with light intensity between 0 to 820 μmol photons m^{-2} s^{-1}.

Biocompatibility Measurements of MPN Nanoshells

The biocompatibility of MPN nanoshells was analyzed by the alamarBlue assay, which is based on the reduction of alamarBlue reagent to highly fluorescent resorufin by metabolically active microalgal cells. Briefly, 100 μL of *E. gracilis* or Eug@MPN cell suspension (5×10^4 cells/well) was incubated with 10 μL of alamarBlue reagent in 96-well plates for 4 h and the fluorescence intensity of cell suspension was measured with excitation wavelength at 540 nm and emission wavelength at 590 nm.

For live/dead cell staining assay, Eug@MPN were stained with a mixture solution comprising FDA (120 μM) and PI (4.5 μM) for 20 min in dark. Stained Eug@MPN were observed with an inverted fluorescence microscope (Zeiss Axio Vert.A1) after washing with H_2O three times. For flow cytometry analysis, Eug@MPN were resuspended in 120 μM FDA solution and incubated for 20 min in dark. After washing with H_2O three times to remove excess FDA, the stained Eug@MPN were analyzed with a flow cytometer (Beckman Coulter CytoFLEX).

Disassembly of MPN Nanoshells on Microalgae

To completely disassemble MPN nanoshells, 0.5 mL of MPN-coated *E. gracilis* cells was incubated in 1 mL of 10 mM HCl or 1 mL of 20 mM EDTA for 60 min at room temperature. After washing with H_2O twice, the *E. gracilis* cells were subjected to EG:JM liquid medium for culture.

Control of Cell Division

After MPN nanoshell formation or disassembly, the cell number of *E. gracilis* or Eug@MPN was adjusted to 1.5 × 10^5 cells/mL with EG:JM medium. Then, 20 mL of cell suspension was transferred into 50 mL Erlenmeyer flasks for growth. The generation time was calculated based on the linear-fitted plot of cell number versus time according to the equation:

$$\text{Generation time} = \frac{t}{3.3 \times \log N_t / N_0} \cdots\cdots (2)$$

where t is the time interval in hours, N_t and N_0 are the number of microalgal cells at the end and beginning of the time interval, respectively.

Measurement of Pigment Yields

Aftter disassembly of MPN nanoshells, *E. gracilis* were resuspended in EG:JM medium for culutre. After 5 days of cultivation, cells were collected and washed with H_2O. Pigments were extracted with 99.9% methanol in dark until the cell pellets were colorless. After centrifugation (5000 *g*, 5 min), the absorbance of the supernatant was analyzed at 665, 652, and 462 nm. The pigment yields were calculated according to the following equations:[3]

$$\text{Chlorophyll a (mg/L)} = 16.72 \times OD_{665} - 9.16 \times OD_{652} \cdots\cdots (3)$$

$$\text{Chlorophyll b (mg/L)} = 34.09 \times OD_{652} - 15.28 \times OD_{665} \cdots\cdots (4)$$

$$\text{Carotenoids (mg/L)} = \frac{1000 \times OD_{462} - 1.63 \times \text{Chlorophyll a} - 104.9 \times \text{Chlorophyll b}}{221} \cdots\cdots (5)$$

Modulation of Cell Motility

After MPN nanoshell formation, 8 µL of Eug@MPN_3 suspension was placed onto a glass slide and left standing for 1 min. Then, HCl (10 mM) was added dropwise next to the cell suspension to initiate the disassembly process of the MPN nanoshells. The disassembly process of MPN nanoshells and the revival of *E. gracilis* motility were recorded by a Nikon ECLIPSE Si microscope.

Protection from Copper-Induced Cytotoxicity Analysis

E. gracilis and Eug@MPN cells (4×10^5 cells/mL) were treated with 0.2 mM $CuCl_2$ in EG:JM liquid medium and incubated for 24 or 48 h under the same condition used for cultivation. After incubation, the treated cells were washed with H_2O three times and the cell viability was investigated by the alamarBlue assay. For fluorescence microscopy analysis, cells were stained with 1 µM SYTOX green for 10 min and observed with an Olympus IX73 inverted fluorescence microscope and confocal microscopy.

Protection from Antibiotic G-418 Analysis

E. gracilis and Eug@MPN cells (2×10^6 cells/mL) were treated with 50 µM antibiotic G-418 in EG:JM liquid medium and incubated for 24 h under the same condition used for cultivation. After incubation, the treated cells were washed with H_2O three times and the cell viability was analyzed by the alamarBlue assay. For fluorescence microscopy analysis, cells were stained with 1 µM SYTOX green for 10 min and observed with an Olympus IX73 inverted fluorescence microscope and confocal microscopy.

Results and Discussion

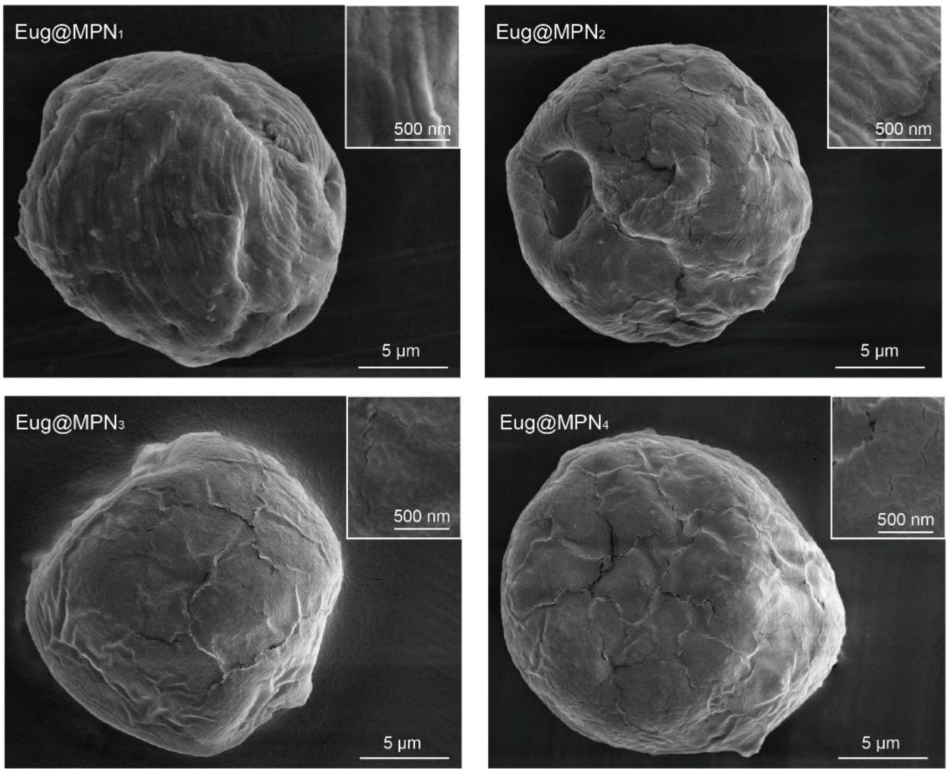

Figure S1. SEM images of Eug@MPN$_1$, Eug@MPN$_2$, Eug@MPN$_3$, and Eug@MPN$_4$.

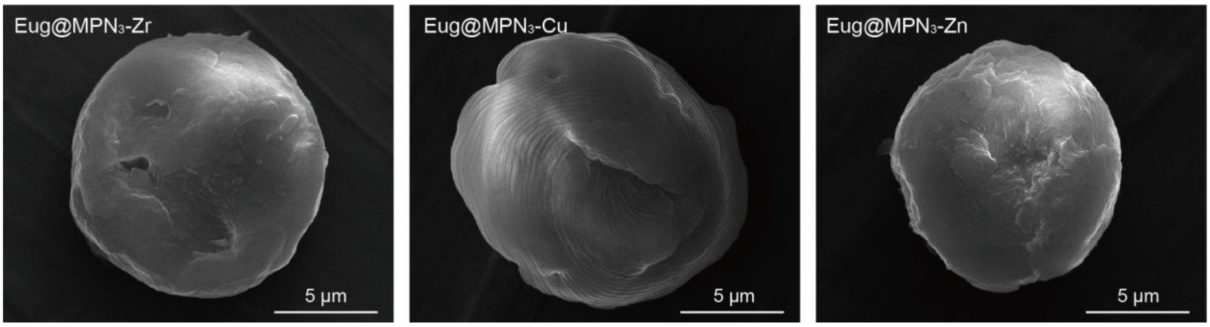

Figure S2. SEM images of *E. gracilis* coated with MPN nanoshells assembled with Zr^{4+}, Cu^{2+}, and Zn^{2+}.

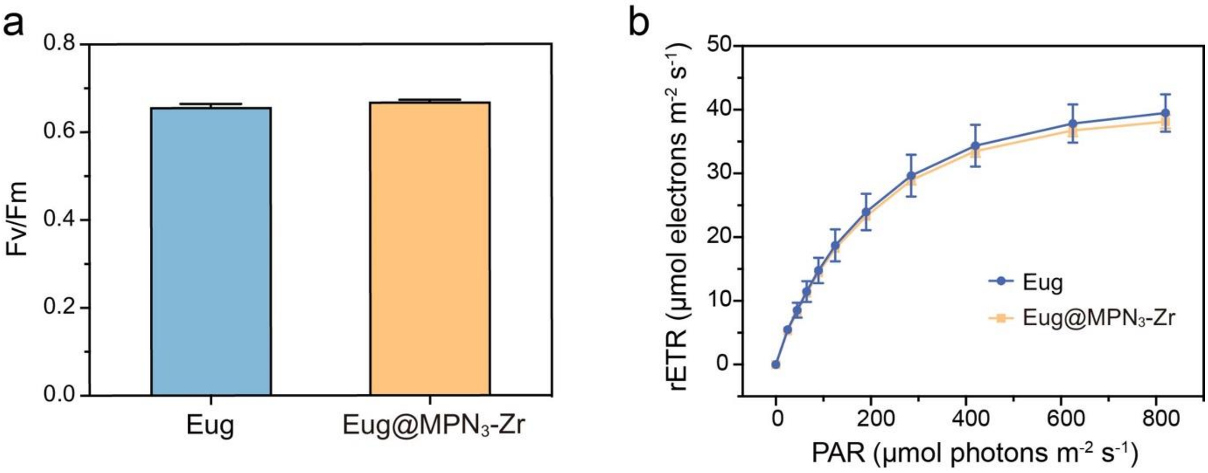

Figure S3. (a) F_v/F_m and (b) rETR of *E. gracilis* and Eug@MPN_3-Zr.

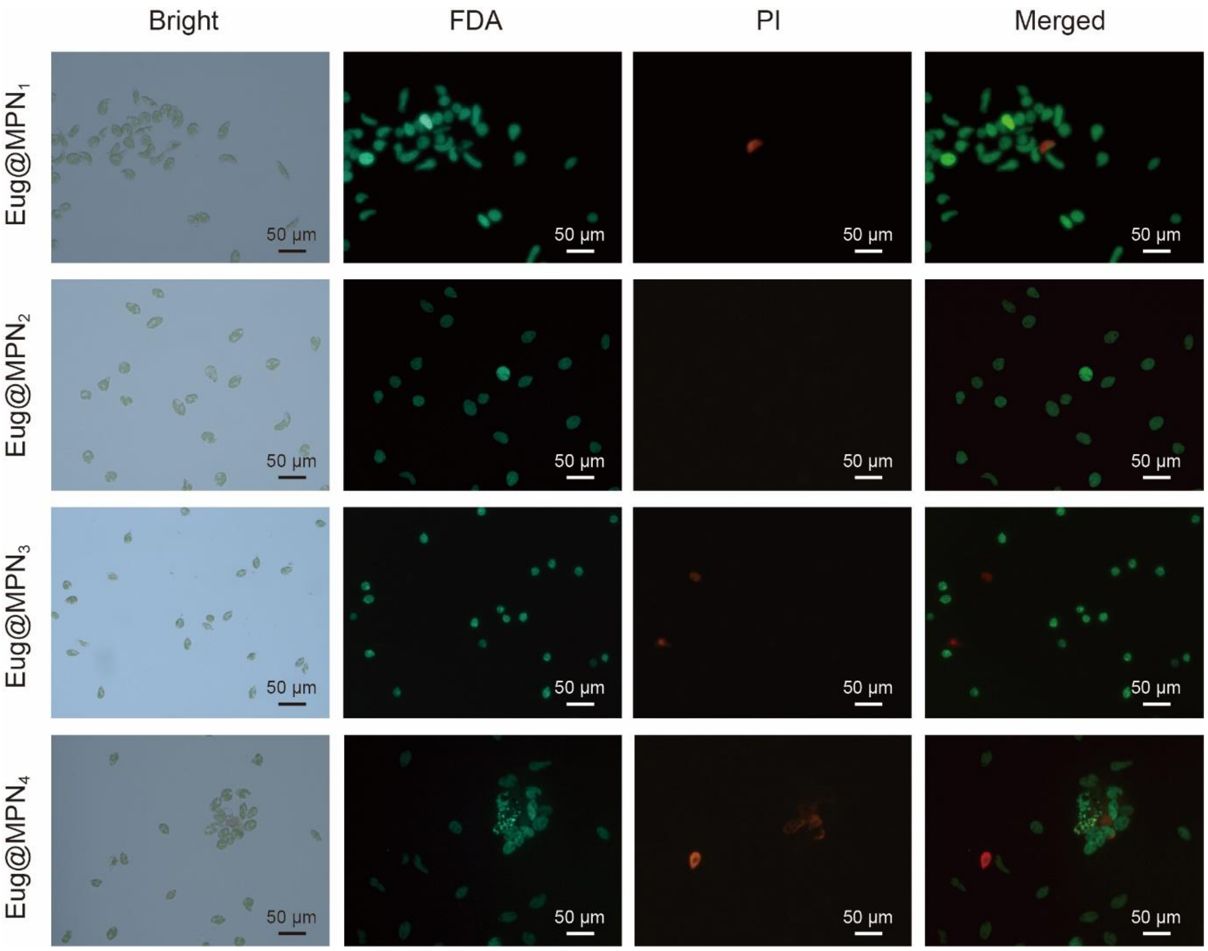

Figure S4. Fluorescence microscopy images of live/dead staining of *E. gracilis* after 1–4 cycles of MPN coating.

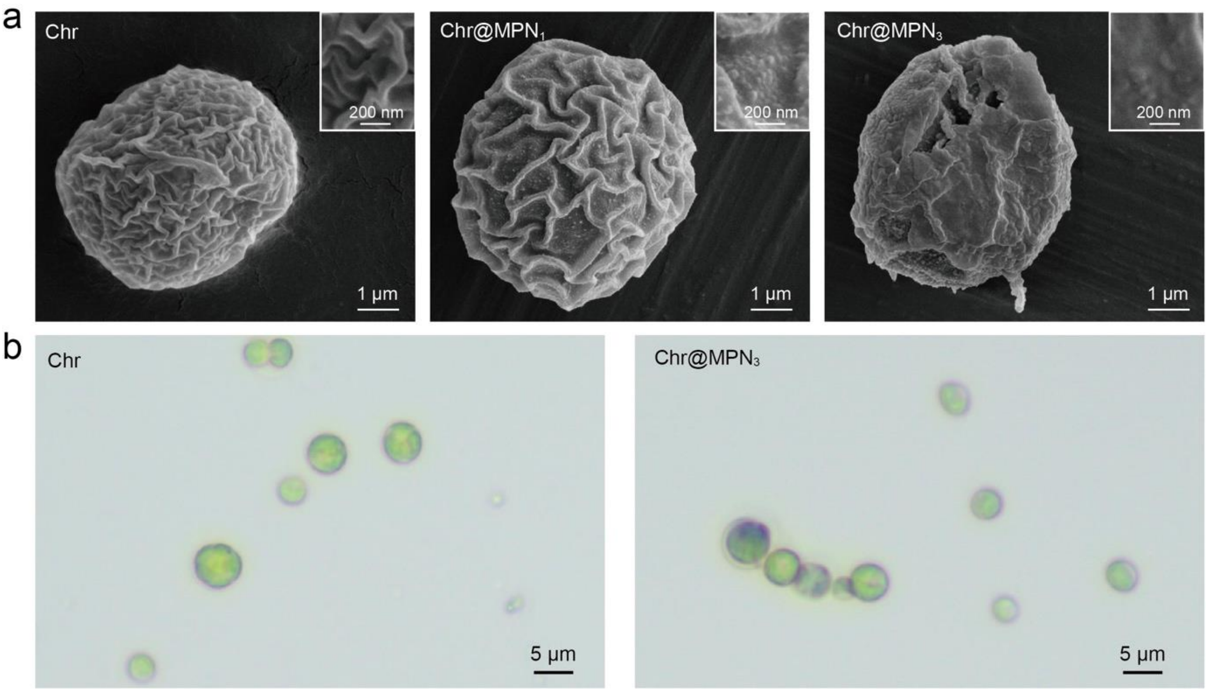

Figure S5. (a) SEM images of *C. zofingiensis*, Chr@MPN$_1$, and Chr@MPN$_3$. (b) Optical microscopy images of *C. zofingiensis* and Chr@MPN$_3$.

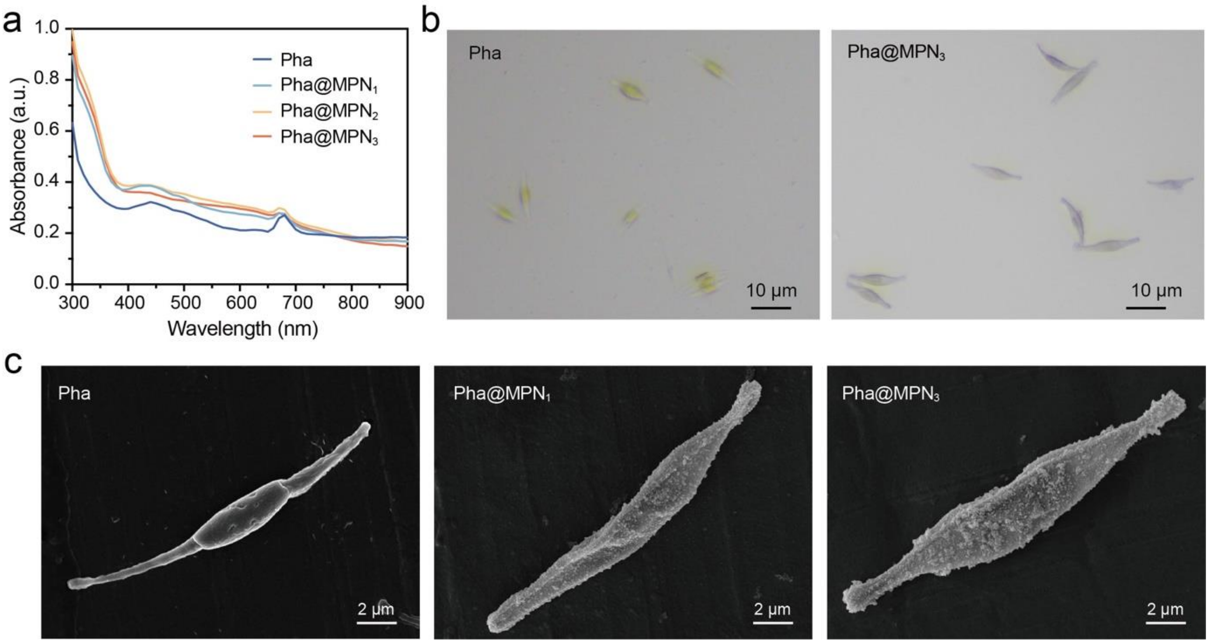

Figure S6. (a) UV–vis absorption spectra of *P. tricornutum* before and after different cycles of MPN coating. (b) Microscopy images of *P. tricornutum* and Pha@MPN$_3$. (c) SEM images of *P. tricornutum*, Pha@MPN$_1$, and Pha@MPN$_3$.

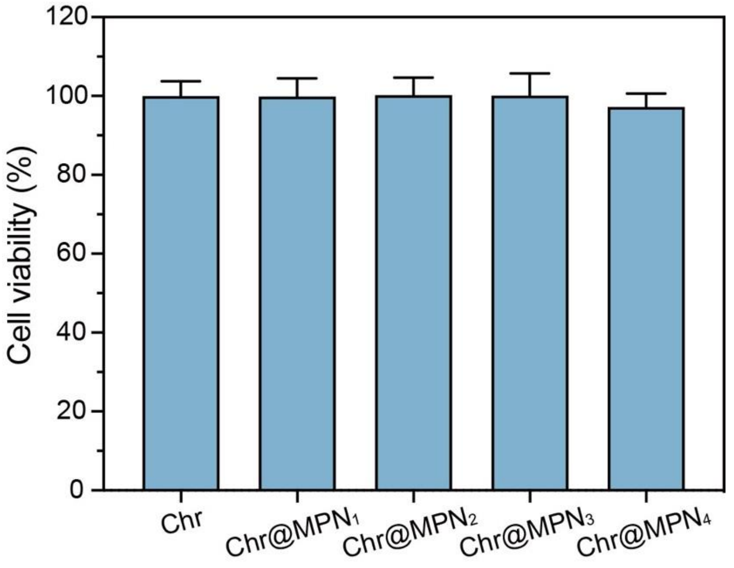

Figure S7. Cell viability of *C. zofingiensis* before and after MPN coating.

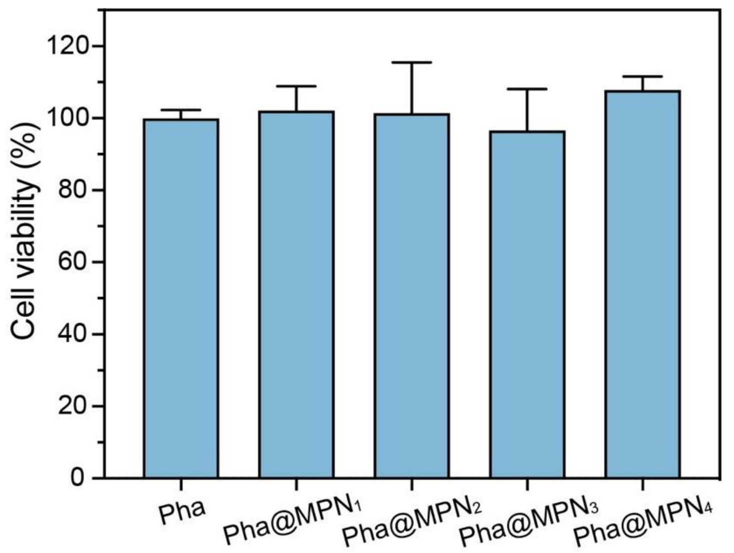

Figure S8. Cell viability of *P. tricornutum* before and after MPN coating.

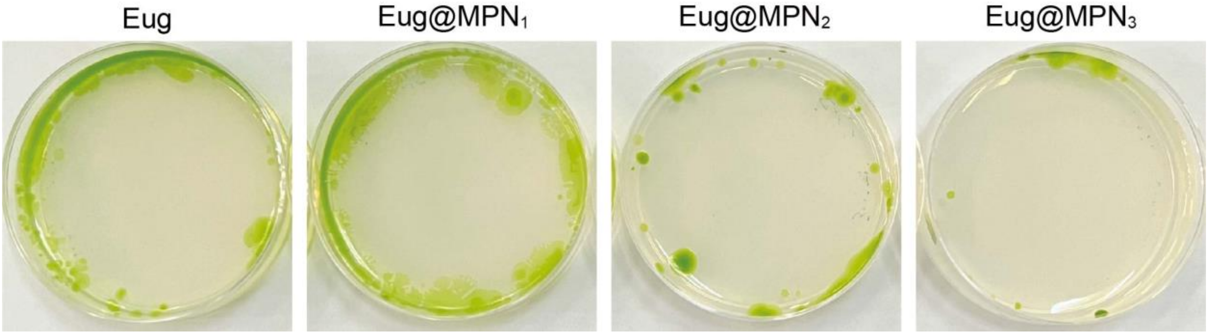

Figure S9. Effects of different layers of MPN nanoshells on the growth of *E. gracilis*. Cells were spread on agar plates and incubated for 10 days.

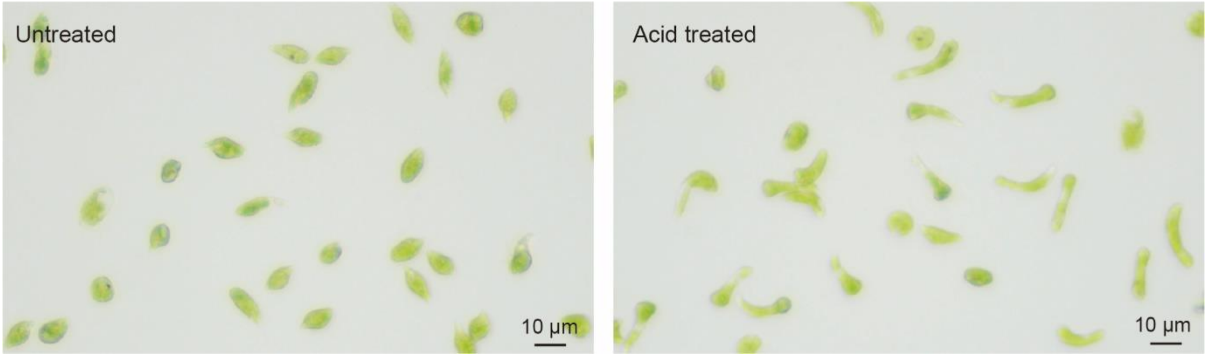

Figure S10. Optical microscopy images of Eug@MPN_3 cells before and after acid treatment for 1 min.

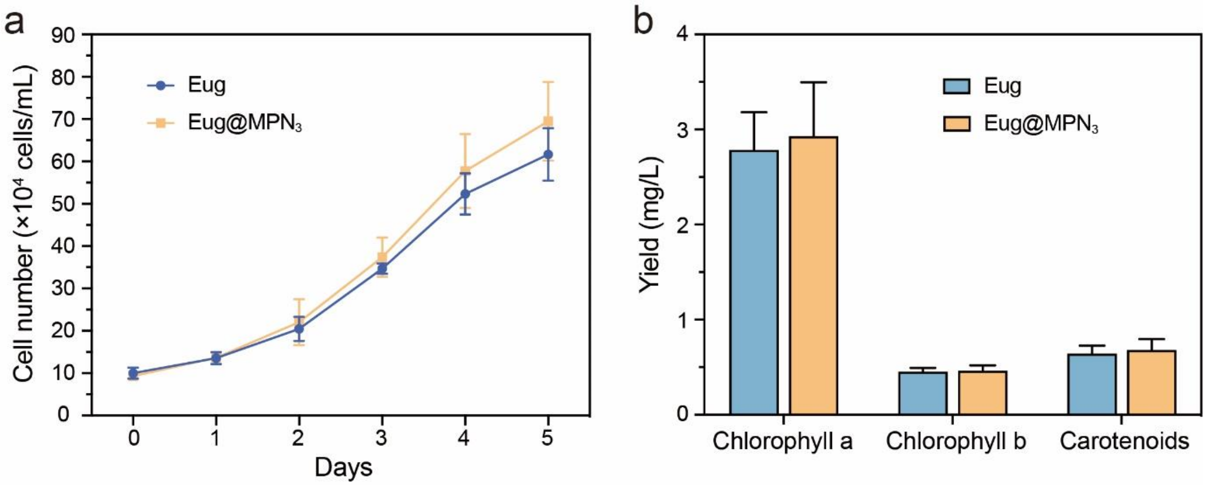

Figure S11. (a) Cell growth profile and (b) yield of chlorophyll a, chlorophyll b, and carotenoids of *E. gracilis* and Eug@MPN$_3$ after disassembly of MPN nanoshells by acid treatment.

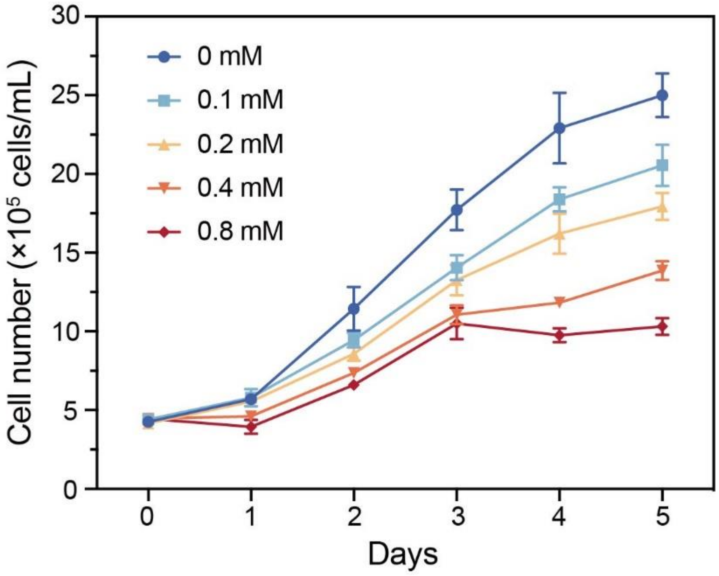

Figure S12. Growth curve of *E. gracilis* cultured with different concentrations of Cu^{2+} for 5 days.

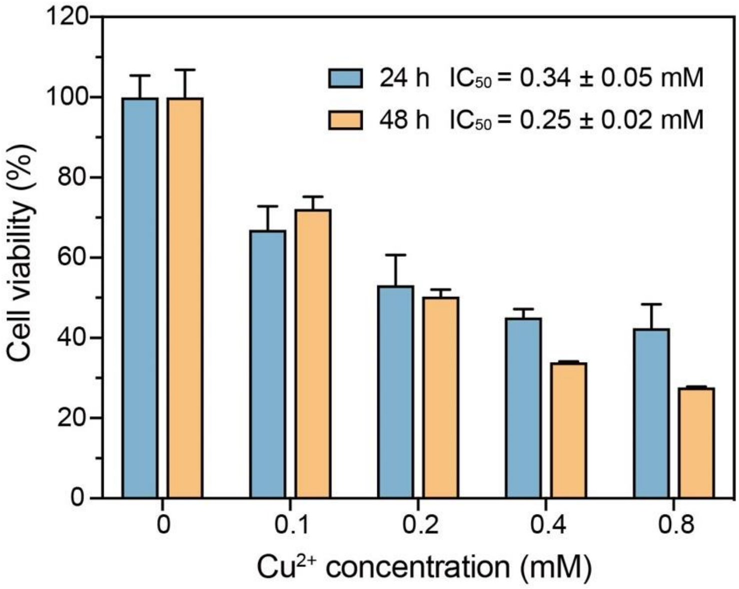

Figure S13. Cell viability of *E. gracilis* treated with different concentrations of Cu^{2+} for 24 and 48 h.

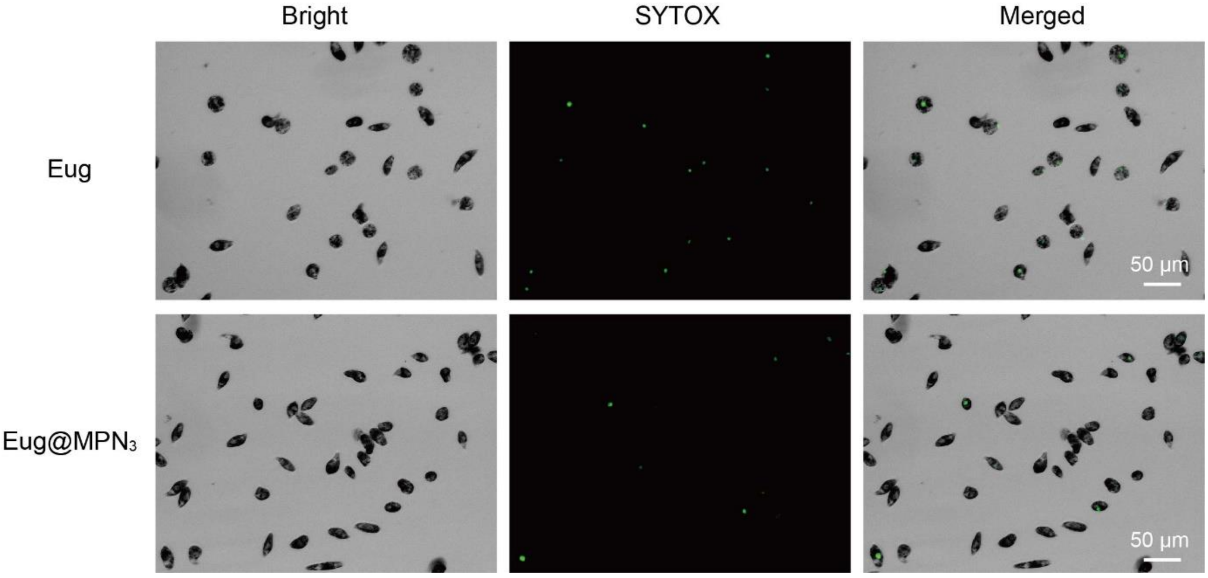

Figure S14. Microscopy images and corresponding fluorescence images of *E. gracilis* and Eug@MPN_3 after culture with 0.2 mM Cu^{2+} for 24 h. The green fluorescence is obtained from dead cells via SYTOX green staining.

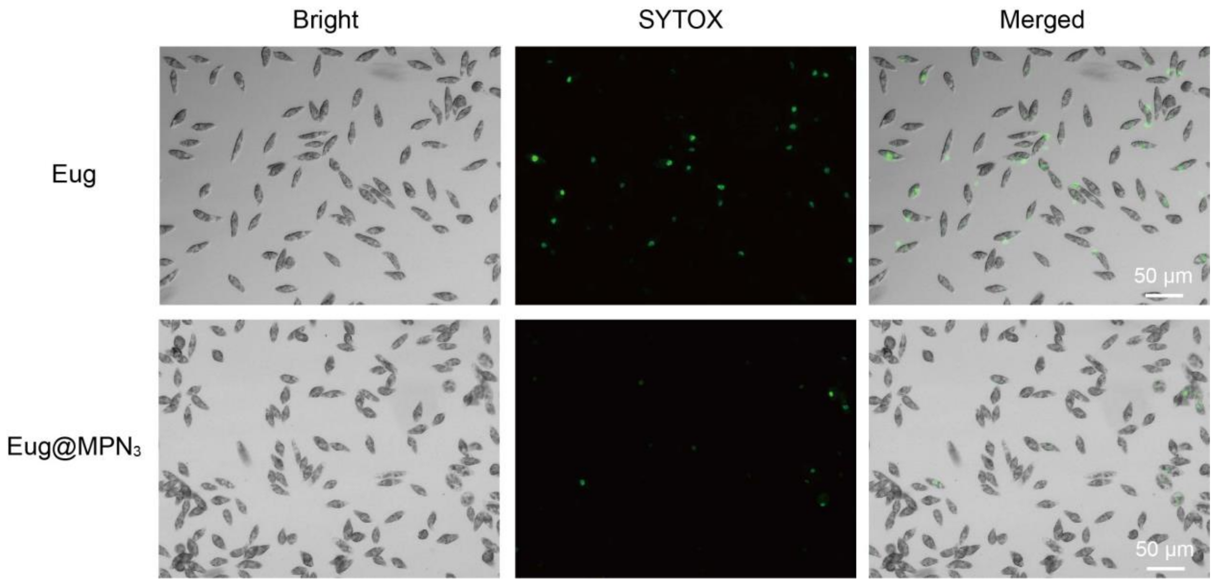

Figure S15. Microscopy images and corresponding fluorescence microscopy images of *E. gracilis* and Eug@MPN$_3$ after culture with 0.2 mM Cu^{2+} for 48 h. The green fluorescence is obtained from dead cells via SYTOX green staining.

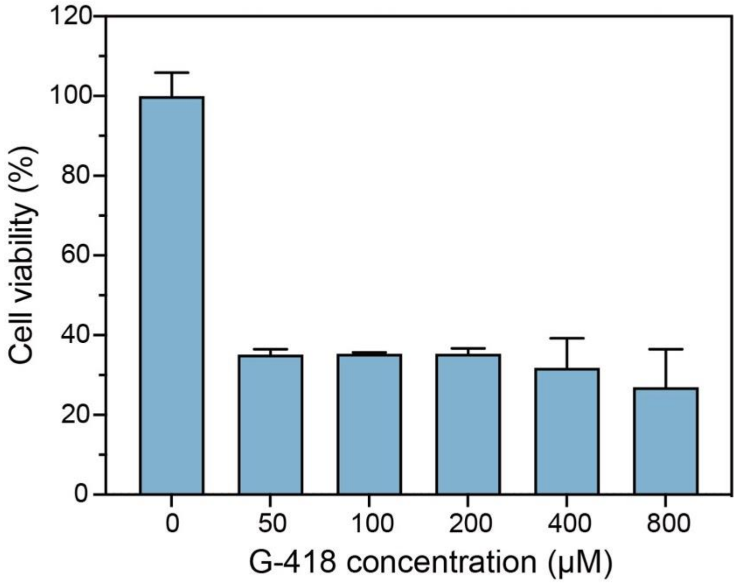

Figure S16. Cell viability of *E. gracilis* treated with different concentrations of antibiotic G-418 for 24 h.

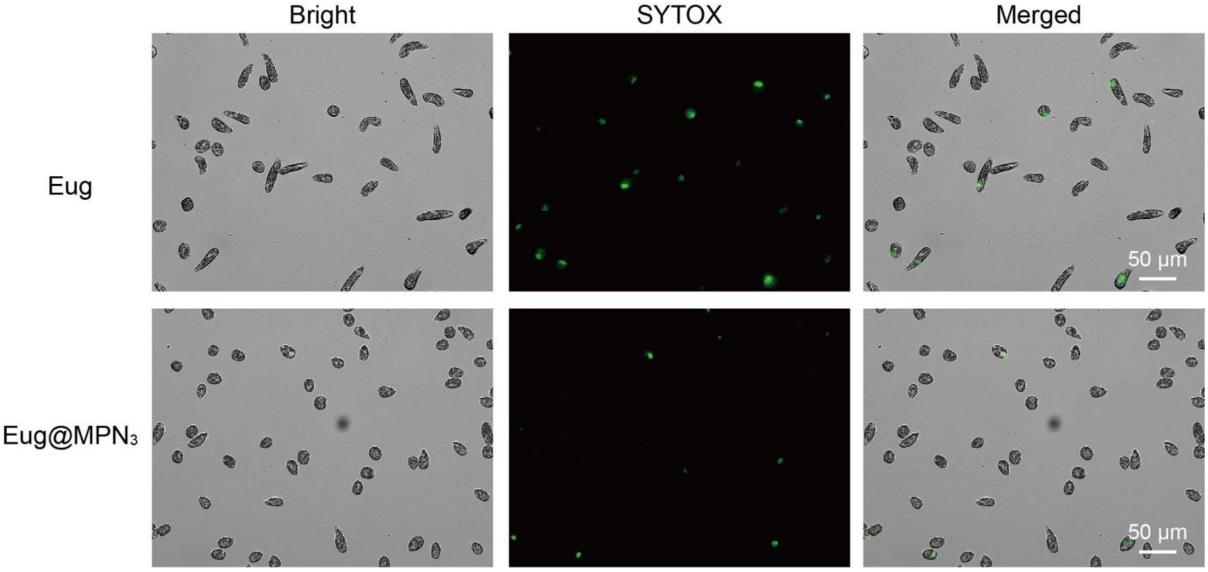

Figure S17. Microscopy images and corresponding fluorescence images of *E. gracilis* and Eug@MPN_3 after culture with 50 μM G-418 for 24 h. The green fluorescence is obtained from dead cells via SYTOX green staining.

Movie S1.
Mobility of *E. gracilis* and Eug@MPN_3.

Movie S2.
Disassembly of MPN nanoshells under acid condition

Movie S3.
Revival of microalgal motion.

.

References

[1] Z. X. Lin, J. J. Zhou, Y. J. Qu, S. J. Pan, Y. Y. Han, R. P. M. Lafleur, J. Q. Chen, C. Cortez-Jugo, J. J. Richardson, F. Caruso, *Angew. Chem. Int. Ed.* **2021**, *60*, 24968-24975.

[2] W. Fu, A. Chaiboonchoe, B. Khraiwesh, M. Sultana, A. Jaiswal, K. Jijakli, D. R. Nelson, A. Al-Hrout, B. Baig, A. Amin, K. Salehi-Ashtiani, *Sci. Adv.* **2017**, *3*, e1603096.

[3] X. J. Li, H. Sun, X. M. Mao, Y. M. Lao, F. Chen, *ACS Sustainable Chem. Eng.* **2020**, *8*, 7600-7608.
